# Epidemiological transition and double burden of diseases in low-income countries: the case of Mozambique

**DOI:** 10.11604/pamj.2020.37.49.23310

**Published:** 2020-09-14

**Authors:** Fausto Ciccacci, Stefano Orlando, Noorjehan Majid, Cristina Marazzi

**Affiliations:** 1 UniCamillus, Saint Camillus International University of Health Sciences, Rome, Italy,; 2 Department of Biomedicine and Control, University of Rome Torvergata, Rome, Italy,; 3 DREAM Program Mozambique, Community of Sant'Egidio, Maputo, Mozambique,; 4 LUMSA, Rome, Italy

**Keywords:** Epidemiological transition, double burden of diseases, Mozambique

## Abstract

Epidemiological transition theory aims to describe changes in epidemiological scenarios at the global and national level. The assumption is the shift from infectious diseases (IDs) to non-communicable diseases (NCDs). Some authors argue that this theory failed to describe epidemiology in sub-Saharan Africa. We considered the case of Mozambique, where is occurring a rapid demographic change, with dramatic growth of the population. According to the data, we concluded that NCDs are increasing in Mozambique, but due to the vast predominance of IDs, a double burden of disease model is more accurate to describe the actual epidemiological context of the country. Consequently, health funding focusing on IDs should take into account the concomitant epidemiological scenario and try to encompass other health challenges.

## Essay

**The epidemiological transition model:** epidemiological transition is a theory that was presented by Omran almost 50 years ago [1]. It aimed to describe significant changes in epidemiological scenario of world populations. Omran studied trends in demographic dynamics in four countries over a more than 150-years period: UK, Japan, Cylon and Chile. He identified three phases of transition: “age of pestilence and famine”, “age of receding pandemics”, and “the age of degenerative and man-made diseases”. Following the demographic transition theory, elaborated at the beginning of the 20^th^ Century [2], the shift from a pattern of disease to another could act as a perfect explanation of these demographic dynamics. The idea was that populations´ health was shifting from a high burden of infectious diseases (IDs) to degenerative and non-communicable diseases (NCDs), with consequences in terms of mortality and fertility rates. Omran himself updated the theory several times [2], but since the first proposition in 1971, it was clear that a general model wouldn´t be complex enough to describe different settings. For that reason, Omran elaborated variants of the model: the classical or Western model (for the US and western Europe), the accelerated version of the classical model (for eastern Europe countries, Japan and Russia), the delayed model (for developing countries), and the transitional variant of the delayed model (for some developing countries with a more accelerated transition, such as China, Taiwan, Sri Lanka) [3]. According to Omran, the change could be faster or slower, but the direction was clear: a shift from IDs to NCDs.

The epidemiological transition model was discussed, criticized, and revised by many authors until recent years [4]. Despite its limitations, Omran´s theory influenced researchers in many fields. Since its first description, the model was applied to various contexts with different results [5]. Many authors identified the need for a more comprehensive theory composed of more consecutive transitions. According to Armelagos and Harper, a first transition should be considered with the shift from foraging to agriculture, consequent urbanization and rise in IDs. A second transition could correspond to the Omran´s one: the drift from IDs to NCDs. The last transition is the one we are entering now: persistence of NCDs, together with the emergence of new or old IDs [6]. In the present document, we will refer to Omran´s theory and the different models as he presented them in 1983 [3]. The attempt to embrace different scenarios is commendable, but it has some significant limits. It is not able to describe epidemiology in some settings, particularly in many sub-Saharan African countries where IDs are still burdening health systems [7].

**Figure 1:**
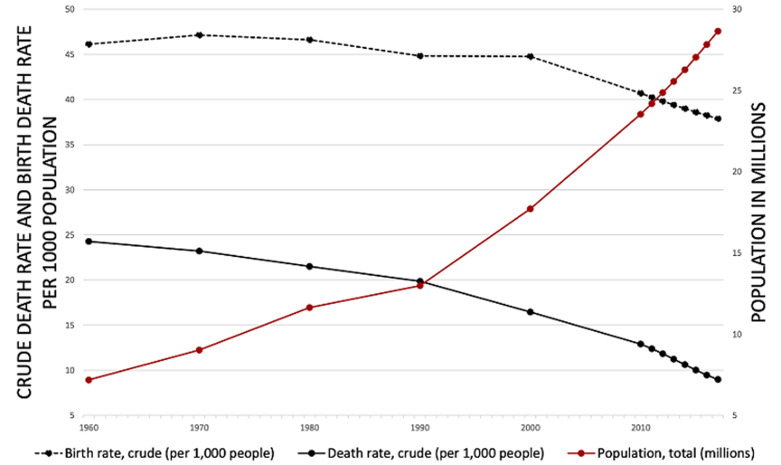
demographic trends in Mozambique 1960-2017 (elaboration of data from World Bank, World Development Indicators, available at https://data.worldbank.org/indicator)

**The epidemiological scenario in Mozambique:** we will review available data about Mozambique and try to evaluate its dynamics in population and epidemiological context. For that purpose we will use data provided by the Global Burden of Diseases (GBD) study [8] and the World Development Indicators published by the World Bank [9]. The GBD study data are freely accessible online, available for worldwide scholars and policymakers [10]. Mozambique is one of the poorest countries in the world ranking 180 out of 189 countries according to the Human Development Index [11]. According to epidemiological transition theory, Mozambique should fit in the “delayed model”: a massive reduction of mortality in the second half of 20^th^ Century following the implementation of western medicine in Africa, together with a persistently high fertility rate [3]. Figure 1 displays trends in crude death and birth rates in the period 1960-2017 together with the population growth. Both mortality and fertility decreased in the last years (respectively, with a 63.1% and 17.9% reduction). These phenomena reflect the current changes in the demography of Mozambique. As some authors already pointed out, African demography has some peculiarities, in particular regarding fertility transition. Bongaarts and Casterline noticed that the decline in fertility as a consequence of socio-economic growth is slower in sub-Saharan countries [12]. This delay could result in a dramatic increase in the African population. Even if Mozambique was thought to have slower growth in population size in comparison with other countries in the area, some authors disagree [13]. World Bank data report an increase in the Mozambican population from 7.2 million in 1960 to 28.6 million in 2017, with the enormous rise of 298,7% (Figure 1). These data seem to support Zinkina and Korotayev considerations.

**Figure 2:**
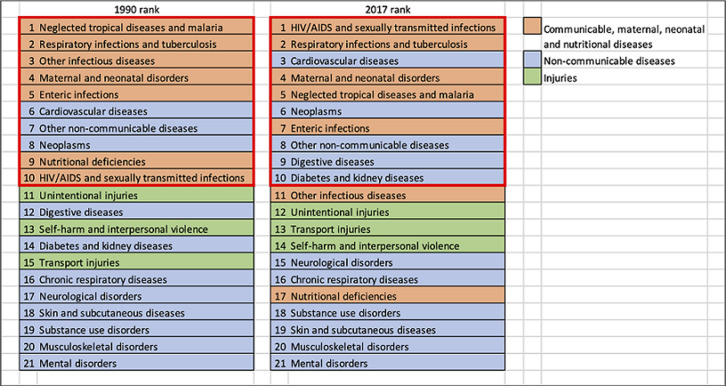
top ranking of causes of death in Mozambique in 1990 and 2017 (data from Global Burden of Diseases, available at https://vizhub.healthdata.org/gbd-compare/)

**Figure 3:**
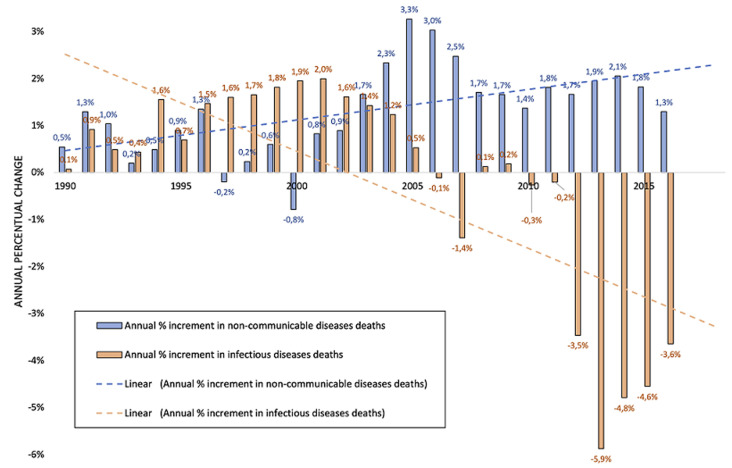
annual percentual change in number of deaths for non-communicable diseases and infectious diseases (elaboration of data from Global Burden of Diseases, available at https://vizhub.healthdata.org/gbd-compare/)

**Figure 4:**
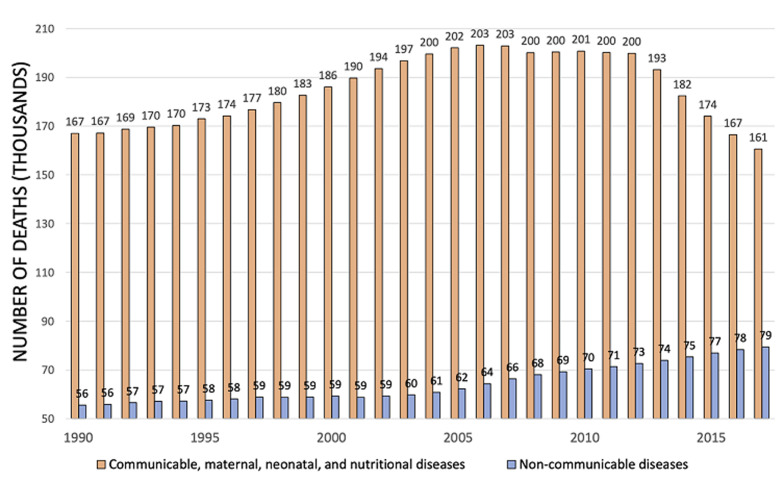
causes of deaths in Mozambique (elaboration of data from Global Burden of Diseases, available at https://vizhub.health data.org/gbd-compare/)

Figure 2 reports the top-ranking causes of death in 1900 and 2017 in Mozambique. IDs are still 4 out of the first five causes of death in the country, even though cardiovascular diseases (CVD) increased. The growth of NCDs in Africa and Mozambique in recent years is indisputable [14,15]. Nonetheless, the dramatic weight of IDs (HIV/AIDS, tuberculosis and malaria in particular) is still high in the country [16]. If we consider the top 10 causes of death in Mozambique in 2017, as displayed in the right side of Figure 2, we notice that 5 are IDs (HIV/AIDS, respiratory diseases and tuberculosis (TB), maternal and neonatal causes, NTDs and malaria) and 5 are NCDs (CVD, neoplasms, other NCDs, digestive diseases and diabetes and chronic kidney disease). These considerations can give a quick perception of what double burden of disease is for Mozambique [17]. Deaths due to NCD are growing each year. Figure 3 shows the annual percentual change in crude deaths rates due to NCDs and IDs; it is clear the growing death toll of NCDs. In any case, if we consider the crude number of deaths for IDs and NCDs, the difference is still huge. As shown in Figure 4, IDs are killing a higher amount of Mozambican every year: around 167.000 deaths in 2017, whereas deaths from NCDs were “only” around 79,300. According to data available in the literature, many IDs are still striking the country [16,18].

The recent spread of SARS-CoV-2 in many low-income countries is worrying. In Mozambique the first case was registered on March 22^nd^ [19], few cases are registered at the moment, but the capacity of the African health system to face a new epidemiological crisis is weak [20]. Moreover, it could be affirmed that the scarce development of the transition is to blamed not on the lack of increment of NCDs, but on the too slow decline in ICs. This factor is driving to the double burden scenario, that could be the more fitting model to describe the epidemiological situation in the country [17]. Mozambique is experiencing a change in demographics dynamics, with dramatic growth in the size of the population. This condition is coherent with other neighbour countries. According to the United Nations (UN), Africa is the fastest-growing continent with more than half of the population increment in the next decades occurring in the continent [21]. This population growth is concerning mainly the cities, with a consequent increase of urbanization. As described by many authors, urbanization is related to behavioural risk factors for NCDs, with a probable effect on the health status of the communities [22,23]. Therefore we can predict that in Mozambique we will assist to growth in NCDs, as it is already happening [15,24]. Although the indisputable increase in NCDs, the massive predominance of IDs suggests that the epidemiological transition is still far to accomplish in the country. In recent years, some authors debated whereas disease-specific funds improved overall health indicators in low-income countries [25,26]. Most of the papers focus on HIV projects in Africa, and authors are not unanimous. In any case, the double burden of disease model indicates that specific funds focusing on IDs should take into account also the concomitant epidemiological scenario. Some researches and public health projects have been implemented in that sense, building up on HIV/AIDS programs to address also NCDs [27,28].

## Conclusion

The epidemiological transition model could hardly predict the evolution of health status in Mozambique as it is primarily related to the national response to IDs. Although the country is experiencing economic growth and demographic transition (even if slow), real socio-economic improvement is not widespread [29,30]. These factors could be responsible for the slowing down of the shift towards NCDs. Due to these considerations, a double burden of disease model would be more accurate to describe the actual epidemiological context of the country. This conclusion has some important implications in terms of public health and health planning. Mozambique gives an example on how the international and national health planning should take into account multiple variables and refer to a complex and variable epidemiological scenario, in order to give proper health responses.

## References

[1] Omran A (1971). The epidemiologic transition: a theory of the epidemiology of populations change. Millbank Memorial Fund Q.

[2] Omran AR (1998). The epidemiologic transition theory revisited thirty years later. World health statistics quarterly.

[3] Omran AR (1983). The epidemiologic transition theory. A preliminary update. Journal of tropical pediatrics.

[4] Santosa A, Wall S, Fottrell E, Högberg U, Byass P (2014). The development and experience of epidemiological transition theory over four decades: a systematic review. Global health action.

[5] Morand OF (2004). Economic growth, longevity and the epidemiological transition. The European Journal of Health Economics. formerly: HEPAC.

[6] Armelagos GI, Harper KN (2005). Disease Globalization in the Third Epidemiological Transition. Globalization, health, and the environment: an integrated perspective.

[7] Sanders JW, Fuhrer GS, Johnson MD, Riddle MS (2008). The Epidemiological Transition: The Current Status of Infectious Diseases in the Developed World versus the Developing World. Science Progress.

[8] Institute for Health Metrics and Evaluation (2018). GBD Compare Data Visualization.

[9] World Bank (2018). GDP per capita, PPP (constant 2017 international $): World Development Indicators Washington D.C.

[10] Institute for Health Metrics and Evaluation (2016). GBD Data.

[11] UNDP (2019). Human Development Report 2019 - Beyond income, beyond averages, beyond today: inequalities in human development in the 21^st^ Century.

[12] Bongaarts J, Casterline J (2013). Fertility transition: is sub-Saharan Africa different?. Population and development review.

[13] Zinkina J, Korotayev A (2014). Projecting Mozambique´s demographic futures. Journal of Futures Studies.

[14] Mudie K, Jin MM, Tan Kendall L, Addo J, Dos-Santos-Silva I (2019). Non-communicable diseases in sub-Saharan Africa: a scoping review of large cohort studies. J Glob Health.

[15] Silva-Matos C, Beran D (2012). Non-communicable diseases in Mozambique: risk factors, burden, response and outcomes to date. Globalization and health.

[16] Berg A, Patel S, Aukrust P, David C, Gonca M, Berg ES (2014). Increased severity and mortality in adults co-infected with malaria and HIV in Maputo, Mozambique: a prospective cross-sectional study. PloS one.

[17] Marshall SJ (2004). Developing countries face double burden of disease. Bull World Health Organ.

[18] Menendez C, Romagosa C, Ismail MR, Carrilho C, Saute F, Osman N (2008). An autopsy study of maternal mortality in Mozambique: the contribution of infectious diseases. PLoS medicine.

[19] World Health Organization (2020). COVID-19 WHO African Region: External Situation Report 04.

[20] Gilbert M, Pullano G, Pinotti F, Valdano E, Poletto C, Boëlle PY (2020). Preparedness and vulnerability of African countries against importations of COVID-19: a modelling study. The Lancet.

[21] (2019). UN. Department of Economic and Social Affairs - Population Dynamics. World Population Prospects 2019. Highlights ST/ESA/SER.A/423.

[22] Dye C (2008). Health and urban living. Science.

[23] Eckert S, Kohler S (2014). Urbanization and health in developing countries: a systematic review. World Health Popul.

[24] Jessen N, Damasceno A, Silva-Matos C, Tuzine E, Madede T, Mahoque R (2018). Hypertension in Mozambique: trends between 2005 and 2015. Journal of hypertension.

[25] Kabatereine NB, Malecela M, Lado M, Zaramba S, Amiel O, Kolaczinski JH (2010). How to (or not to) integrate vertical programmes for the control of major neglected tropical diseases in sub-Saharan Africa. PLoS Negl Trop Dis.

[26] Matsubayashi T, Manabe YC, Etonu A, Kyegombe N, Muganzi A, Coutinho A (2011). The effects of an HIV project on HIV and non-HIV services at local government clinics in urban Kampala. BMC International Health and Human Rights.

[27] Ciccacci F, Tolno VT, Doro Altan A, Liotta G, Orlando S, Mancinelli S (2019). Non communicable diseases burden and risk factors in a cohort of HIV+ elderly patients in Malawi. AIDS Res Hum Retroviruses.

[28] Njuguna B, Vorkoper S, Patel P, Reid MJA, Vedanthan R, Pfaff C (2018). Models of integration of HIV and noncommunicable disease care in sub-Saharan Africa: lessons learned and evidence gaps. AIDS (London England).

[29] Cunguara B, Fagilde G, Garrett J, Uaiene R, Headey D (2012). Growth without change? A case study of economic transformation in Mozambique. Journal of African Development.

[30] Macuane JJ, Buur L, Monjane CM (2018). Power, conflict and natural resources: The Mozambican crisis revisited. African Affairs.

